# Regulation of the polo kinase during cytokinesis

**DOI:** 10.18632/oncotarget.4885

**Published:** 2015-07-17

**Authors:** David Kachaner

**Affiliations:** Institut de Recherche en Immunologie et en Cancérologie, Université de Montréal, Montréal, Québec, Canada

Mitosis and cytokinesis allow faithful distribution of the duplicated genetic material from one mother to two daughter cells. These cell cycle events are regulated precisely by several kinase families including Cyclin-dependent kinases (CDKs), Aurora kinases and Polo-like kinases (Plks). Interestingly, these proteins are able to collaborate with each other during cell division and defects in their regulations could lead to aneuploidy.

Discovered in Drosophila and conserved from yeast to humans, the Polo kinase has emerged as a potent regulator of cell division [1]. The Polo kinase is the founding member of the Plk family [2]. While budding yeast and fission yeast each have a single Plk (Cdc5 and Plo1 respectively), mammals have five Plk members (Plk1-5). Apart from Polo, the fly has only one additional member named Sak, which is orthologous to Plk4 and appears dedicated to the regulation of centriole duplication. The fly Polo kinase, the single yeast Plks and human Plk1 all promote similar functions during cell division, including mitotic entry, centrosome maturation, spindle assembly, kinetochore function and cytokinesis.

Plks share a conserved amino-terminal Ser/Thr kinase domain (KD) and a carboxy-terminal Polo-Box Domain (PBD) [3]. The PBD acts as a phosphopeptide-binding domain and allows Polo to bind substrates and adaptor proteins that recruit Polo to its subcellular locations including centrosomes, kinetochores and the central spindle during mitosis and the midbody during cytokinesis.

The multiple functions of the Polo kinase are possible because this protein is submitted to a complex regulation. Amongst other modes of regulation, Plks are activated by phosphorylation of their activation loop in the KD [2]. In humans, this activation of Plk1 begins in G2 and is mediated by the Aurora A kinase, whereas in flies the activation of Polo in early mitosis on kinetochores requires its phosphorylation by Aurora B, a subunit of the Chromosomal Passenger Complex. The activity of Polo has long been known to be essential for cytokinesis [4]. However, how Polo is regulated in this process was unknown.

**Figure 1:**
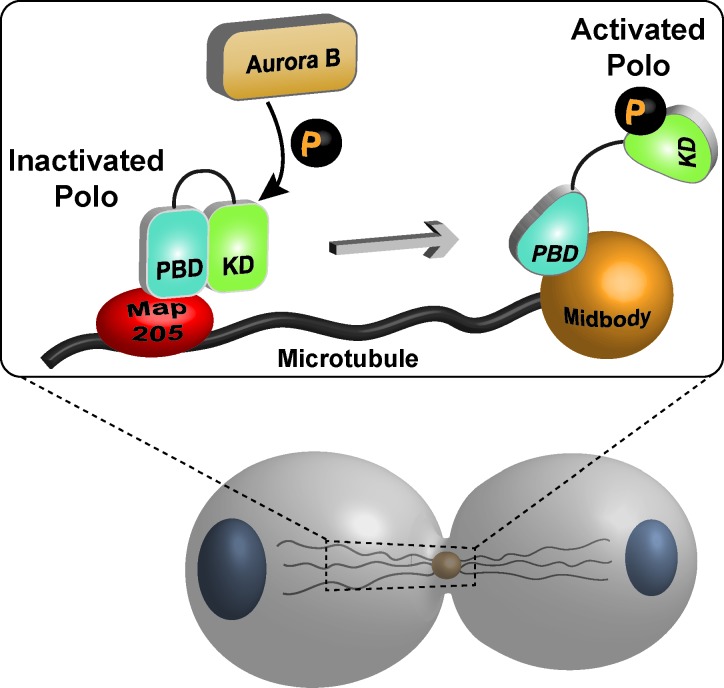
Model for the regulation of Drosophila Polo during cytokinesis Map205 binds and inhibits Polo on microtubules. Activating phosphorylation of Polo by Aurora B during cytokinesis induces its dissociation from Map205 and allows Polo to relocalize to the midbody.

In a recent paper from our laboratory, we have investigated this question in Drosophila cells in culture. We found that Polo phosphorylation by Aurora B is required for successful cytokinesis [5]. This phosphorylation occurs in the activation loop of the KD at Thr182. Our results indicate that upon phosphorylation, Polo dissociates from (or is prevented from binding to) the PBD-bound Map205, a microtubule-associated protein which sequesters unphosphorylated Polo on microtubules and inhibits its kinase activity [6] (Figure 1). Then, freed from this inhibitor, activated Polo is recruited to the midbody. To determine the physiological importance of Polo phosphorylation during cytokinesis, we performed time-lapse microscopy in cells where Aurora B is inhibited or when endogenous Polo was replaced with a nonphosphorylatable form (Polo T182A) fused to the GFP. In both cases, Polo failed to localize properly to the midbody and more than 80% of the cells attempting division failed cytokinesis and became binucleated.

Our findings can be interpreted in structural terms in the light of the recent publication by Xu *et al.* which solved the cocrystal structure of a fragment of Drosophila Map205 in complex with the separate PBD and KD of zebrafish Plk1 [7]. This work reveals that Map205 binds and stabilizes the closed conformation of the PBD, which in turn binds and stabilizes the inactive conformation of the KD. Our findings now suggest that during cytokinesis, phosphorylation on the Polo kinase at its activation-loop negatively regulates the interaction between Polo and its PBD-bound inhibitor Map205 by an interdomain allosteric mechanism and induces a conformational change that is required for full activation of the kinase.

Drosophila Map205 is poorly conserved in vertebrates, but the observation that human Plk1 is competent for an inhibiting interaction with Map205 suggests that there may exist other physiological interactors of Plk1 that regulate it by a similar mechanism. Such proteins remain to be identified.
